# Inter-rater reliability of the neonatal adverse event severity scale using real-world Neonatal clinical trial data

**DOI:** 10.1038/s41372-021-01164-w

**Published:** 2021-09-14

**Authors:** Tamorah Lewis, Norma Terrin, Jonathan Davis, Kurt Michels, Thomas Salaets, Kelly Wade, International Neonatal Consortium

**Affiliations:** 1 Children’s Mercy Hospital, University of Missouri Kansas City School of Medicine, Department of Pediatrics, Kansas City, MO USA; 2 Institute for Clinical Research and Health Policy Studies, Tufts Medical Center, Boston, MA USA; 3 Tufts Children’s Hospital, Tufts University School of Medicine, Department of Pediatrics, Boston, MA USA; 4 Critical PATH Institute, Tucson, AZ USA; 5 Department of Development and Regeneration, KU Leuven, Herestraat 49, Leuven, Belgium; 6 Department of Pediatrics, University of Pennsylvania Perelman School of Medicine, Philadelphia, PA USA

**Keywords:** Translational research, Paediatrics

## Abstract

**Objective:**

The Neonatal Adverse Event Severity Scale (NAESS) was developed to improve scoring of neonatal adverse events (AEs) and accelerate neonatal drug development. This is the first validation study of the novel tool.

**Study design:**

Retrospective validation study assessing the inter-rater reliability (IRR) of the NAESS. Reviewers used real-world AE data from a neonatal trial. Intra-class correlation (ICC) statistical analysis was performed.

**Result:**

Sixty AEs were randomly assigned to twelve reviewers for a total of 240 severity scores. Generic and AE-specific NAESS tables were assessed. The ICC was 0.63 (95% confidence interval 0.51 to 0.73). Percent variation due to reviewer and residual error was 0.03 and 0.34, respectively.

**Conclusion:**

In this first study of the NAESS tool, an ICC of 0.63 indicates moderate reliability. Results highlight the need for improved data collection on neonatal AE forms, augmented training on the NAESS tool, and will inform the prospective validation studies.

## Introduction

Adverse event (AE) severity scoring is an essential component of all therapeutic intervention trials. An AE is defined by the Food and Drug Administration (FDA) as “any untoward medical occurrence associated with the use of a drug in humans, whether or not considered drug related.” AE scoring typically involves assigning a severity grade between 1 (mild) and 5 (death). Events are additionally classified as serious (SAE) if they result in: inpatient hospitalization, prolongation of hospital course, a life-threatening occurrence, or death.

The recognition and classification of AEs is challenging in neonates, particularly those receiving intensive care who are most likely to be involved in clinical trials. Hospitalized neonates can have very complex physiology with multi-organ system dysfunction and many AEs. The unique types of neonatal AEs do not mirror the symptom classification in adults and children. Until recently, there were multiple tools used to assess AE severity in adults and children [1, 2], but none were developed and validated specifically for neonates. Additionally, each pharmaceutical company and clinical research organization use their own approach and interpretation of neonatal AEs, making safety results from different studies difficult to compare. To study and ensure safety of a drug in neonates, investigators need a tool for improvement and standardization of neonatal AE collection and severity classification.

In 2019, the Neonatal AE Severity Scale (NAESS V.1.0) was published and the tool was placed on-line (https://evs.nci.nih.gov/ftp1/INC/Adverse_Events_Terminology/) [3]. This tool was created using a stepwise consensus approach and multiple rounds of revision based on the Delphi approach among diverse stakeholders including physicians, nurses, academic researchers, as well as industry representatives. The NAESS provides a generic table and 35 multiple organ system and AE-specific tables. The NAESS defines severity grade of AEs using suitable neonatal specific severity markers, descriptions, and physiologic parameters. These NAESS terms were linked to lowest level terms in MedDRA and when possible, definitions for AEs were based on the NCI thesaurus Pediatric AE terminology subset [1]. Age-appropriate activity, basal physiology, and care changes are integral indicators of neonatal AE severity in NAESS. While the International Neonatal Consortium (INC) had multi-stakeholder engagement in the creation of this new tool, validation testing using an independent cohort of neonatal AEs was needed in order to examine the performance of the tool in “real-world” situations.

The validation process requires a multi-step procedure to assure internal validity and generalizability. The main goal of a tool for standardization is to reduce interobserver variability. Evaluation of the ability of the tool to improve interobserver agreement is thus a critical aspect of validation. This retrospective study uses real neonatal AE data collected as part of a recently published randomized, controlled trial involving intratracheal administration of a recombinant human club cell (CC10) protein in premature neonates to prevent respiratory morbidity [4]. This approach provided the first step in validation of the NAESS tool by assessing the inter-rater reliability of AE scoring.

## Methods

This retrospective validation study was designed to evaluate inter-rater reliability using the intra-class correlation (ICC) as the statistical test among AE severity scores obtained from different reviewers using the NAESS scoring tool. The study involved 12 independent reviewers and 60 previously reported AEs (referred to as “cases”; 35 SAE and 25 AEs), collected as part of the previously described neonatal clinical trial [4]. Each reviewer assigned severity scores for 20 cases with each case reviewed by four reviewers. This study was submitted to the Children’s Mercy Hospital IRB and was deemed exempt on 3 December 2019. While the study was deemed exempt from IRB review, the invitation to the reviewers included a clear acknowledgment of the following items: (1) the project was identified as research, (2) participation was voluntary, (3) benefits and risks to reviewers were disclosed and (4) contact information of the PI was provided.

### Adverse event data

**Fig. 1:**
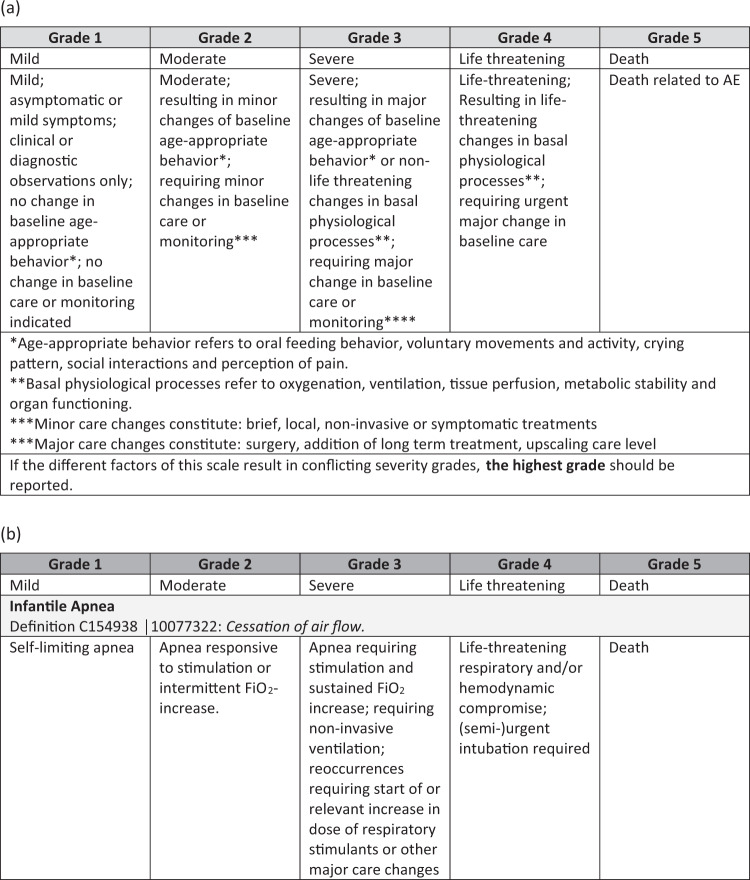
Tables from the Neonatal Adverse Event Severity Scale. Example of **a** generic and **b** AE-specific table. The AE-specific table displayed is for Apnea.

The case data used to assess inter-rater reliability were electronic files of the actual AE forms collected during the following clinical trial [4]. This study assessed the safety and efficacy of a single intratracheal dose of rhCC10 delivered in the first 24 h after premature birth in reducing chronic pulmonary insufficiency of prematurity at 12 months of life. Eighty-eight infants (gestational age 24–29 weeks, birth weight 600–1200 g) were randomized at six study sites. AEs were recorded on standard paper case report forms, identifying information was redacted, and the information was provided to the reviewers. The clinical trial data included a total of 190 cases that were originally reported to be either serious (SAE, *N* = 162) or non-serious (AE, *N* = 28). To select the 60 most representative cases, we a priori chose to identify 35 SAEs and 25 AEs to represent the NAESS spectrum of grades 1–5. Thirty cases were highlighted using disease specific NAESS tables and 30 cases using the generic NAESS table (Fig. 1). The research team used the following case inclusion criteria: (1) the case occurred during the initial birth hospitalization and before the neonate reached a post-menstrual age of 44 weeks; (2) no more than one SAE and one AE was used from each individual neonate; (3) cases were selected to represent physiologic abnormalities covering multiple organ systems and representative of common pathologies in preterm neonates; (4) AE case report form handwriting was legible. Only one death was included among the cases for the validation as it was assumed that there would be no disagreement among reviewers in assigning the correct grade (grade = 5 for a death). We also wanted to focus on the reliability of the NAESS when applied to cases that are more challenging to grade.

### Case reviewers

Twelve independent AE reviewers were asked to assign severity scores for the cases. The study team solicited interested participants from among members of INC. We excluded participants who were directly involved in the development of the original NAESS tool. Among interested participants, 12 reviewers were chosen who had previous experience with neonatal clinical trials and represented different neonatal clinical trial stakeholders (academia, regulators, industry). The case assignment randomization was stratified by whether the case was deemed in the original clinical trial to be an SAE versus AE, whether there existed a specific NAESS table corresponding to it, or whether the generic table was required.

Each reviewer received a standard zip file by email that included the NAESS tool (including the generic table and the 35 published AE specific tables), 20 individual de-identified case-report forms for the specific case; and an excel file for recording demographic data pertaining to their trial experience and the severity score (#1–5) that was assigned. The pre-defined severity assessment from the original RCT was masked in the file naming. The reviewer was blinded as to whether the case was deemed an SAE or AE in the original RCT, but the file name did specify “0” for generic table use and “1” for specific table use.

### Sample size and statistical analysis

A sample size of 60 cases with 4 independent reviews per case was projected to result in a 95% confidence interval for the intra-class correlation coefficient (ICC) of width 0.28, 0.26, 0.19, or 0.08 if the true ICC were 0.3, 0.5, 0.7, or 0.9, respectively. Data were analyzed using a linear mixed effects model fit by restricted maximum likelihood with random effects for case and reviewer. The percent of variation attributable to case, reviewer, and error were calculated from the model. The ICC is the percent of variation attributable to the case and is used as a measure of inter-rater reliability. Analyses were repeated, subgrouping the cases by serious versus non-serious status and whether all reviewers used matching NAESS tables. R version 3.6.0 and packages lme4 and irr were used for calculations http://www.R-project.org/ [5, 6].

## Results

This study explored AE severity scores among a broad range of adverse clinical trial events, both SAEs and AEs, utilizing disease-specific and generic tables found in the NAESS scoring tool (Table 1). Reviewer’s neonatal clinical trial experience is depicted in Table 2. The reviewers were mostly from academia and government including principal investigators, research coordinators, a data safety monitoring board member, and a regulator (Table 2).

**Table 1:** List of NAESS tables used by reviewers to define severity to cases.

*Name of NAESS scoring table*	Serious adverse event	Adverse event
AEs category as defined by original RCT	35	25
Generic NAESS table^a^	9	12
Infectious—Neonatal Culture Positive Sepsis	5	0
Neurological—Neonatal Intraventricular Hemorrhage (IVH)	3	3
Respiratory—Infantile Apnea	2	3
Infectious—Neonatal Culture Negative Sepsis	2	1
Neurological—Retinopathy of Prematurity (ROP)	2	1
Respiratory—Neonatal Respiratory Insufficiency	2	1
Gastro-intestinal—Neonatal Gastrointestinal Bleeding	2	0
Gastro-intestinal—Feeding Intolerance	1	1
Respiratory—Neonatal Pulmonary Hemorrhage	1	1
Cardiovascular—Neonatal Coagulation Disorder	1	0
Cardiovascular—Neonatal Edema	1	0
Cardiovascular—Neonatal Tachyarrhythmia	1	0
Gastro-intestinal—Neonatal Spontaneous Intestinal Perforation (SIP)	1	0
Gastro-intestinal— Necrotizing Enterocolitis (NEC)	1	1
Respiratory—Neonatal Pneumothorax	1	0
Cardiovascular—Neonatal Hypotension	0	1
Gastro-intestinal—Necrotizing Enterocolitis (NEC)	0	1

^a^Cases where the generic table was used included PDA, pneumonia, UTI, feeding intolerance, hepatic bleeding, conjunctivitis, inguinal hernia, anemia, thrombocytopenia, hyperbilirubinemia.

The AE-specific tables are listed in descending order by frequency of use.

**Table 2:** Case reviewer demographics.

	Role	Years*	Primary Job Area	Neonatal RCT with any drug^#^	If yes, how many?	Country
**Reviewer 1**	Research Coordinator	20	All^$^	Yes	2	USA
**Reviewer 2**	Research Coordinator	5	Academia	Yes	3	USA
**Reviewer 3**	PI	4	Academia	Yes	4	USA
**Reviewer 4**	PI	2	Academia	No		USA
**Reviewer 5**	PI	16	Academia	Yes	5	USA
**Reviewer 6**	PI	15	Government	Yes	3	UK
**Reviewer 7**	PI	25	Government	Yes	25	UK
**Reviewer 8**	Data Safety Monitoring Board member	3	Academia	Yes	12	USA
**Reviewer 9**	Regulatory Reviewer	3	Government	No		Canada
**Reviewer 10**	Regulatory Reviewer	19	Government	No		USA
**Reviewer 11**	Research Coordinator	12	Academia	Yes	3	USA
**Reviewer 12**	Regulatory Reviewer	20	Academia	No		Japan
**Summary statistics (median, IQR)**		(12, 14)			(3.5, 3.75)	

^$^We believe this response indicates that the reviewer has held jobs in academia and industry.

^*^Number of years in their current job position.

^#^Involvement with neonatal RCT involving a drug within the past three years.

As a result of the randomization, each of the 12 reviewers received 10 events that could be scored using a disease-specific NAESS table (3 AEs and 7 SAEs) and 10 events that could be scored using a generic (non-disease oriented) NAESS table (4 or 5 AEs and 5 or 6 SAEs). The final analysis data set contained severity scores provided by 12 reviewers for 240 events (60 cases, each reviewed 4 times).

**Fig. 2:**
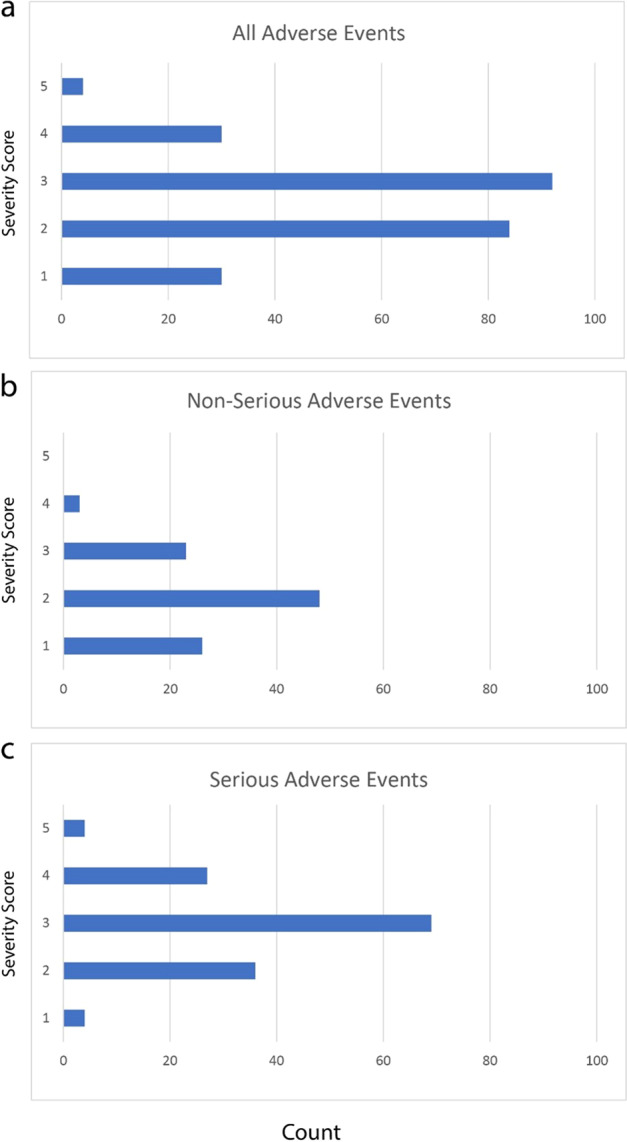
The frequency of severity scores assigned to cases. Panels **a** all 60 cases, **b** cases defined as AEs, and **c** cases defined as SAEs.

**Fig. 3:**
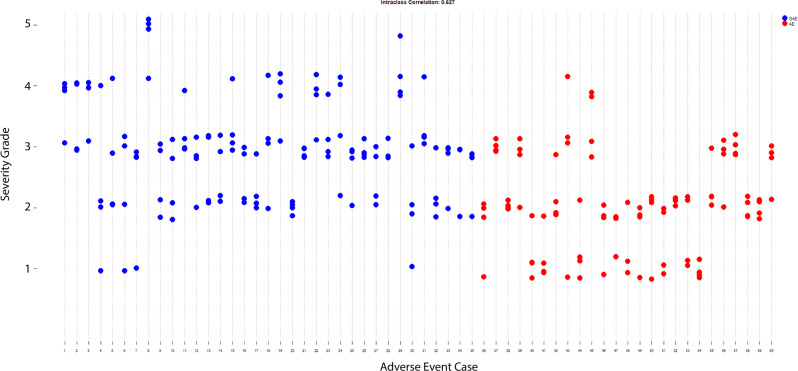
Intra-class correlation of the scores assigned to the 60 cases, each reviewed four times. The *x*-axis is each individual case. Perfect inter-rater reliability is when all four reviewers scored the case the same. Blue dots represent serious adverse events and red dots are non-serious adverse events.

The distribution of the 240 rater-assigned severity scores is seen in Fig. 2. The intra-class correlation (ICC, the percent of total variation due to the cases) was 0.63 (95% confidence interval 0.51 to 0.73). The ICC is graphically displayed in Fig. 3. The percent variation due to reviewer and residual error was 0.03 and 0.34, respectively. The ICCs for the separate analyses of AEs and SAEs were 0.72 (95% CI 0.56 to 0.85) and 0.55 (95% CI 0.39 to 0.71), respectively. When all four raters used the exact same NAESS table to score the AE, the ICC was 0.63 (95% CI 0.487 to 0.758) and when the raters used at least one different table, the ICC was 0.558 (95% CI 0.340 to 0.762). The distribution of the 240 scores can be seen in Fig. 2.

Among the 60 cases, the severity scores that the reviewers returned were completely concordant (same score from all four reviewers) for 11 (18.3%) cases. There were two different severity scores for 43 (71.7%) cases, and three different severity scores assigned for 7 (11.7%) cases. Most case reviews used the disease specific or generic NAESS tables based on the names of the pre-specified tables in the tool. Among the 120 reviews of the 30 cases that the study team had identified to have a matching pre-specified AE-defined NAESS table, the reviewers used the predicted NAESS table in 107/120 (87%) of case reviews. Reviewers unexpectedly chose to use the generic NAESS table in 12/120 (10%) of these cases and a different AE-specific table in 1 case review (coagulation disorder table used instead of intraventricular hemorrhage table). Among the 120 reviews of the 30 cases requiring use of the generic NAESS table, 110/120 (92%) used the generic table. However, 10/120 (8%) reviews were performed using an AE-specific NAESS table. An example of this is a reviewer who used the “respiratory insufficiency” table for review of an AE related to a patent ductus arteriosus (PDA).

Among the 12 reviewers, 10 used a different table than expected at least once. Six reviewers failed to use a corresponding disease-specific table at least once, and five reviewers used an AE-specific table when they should have used the generic table, at least once. Much of the discrepancy in NAESS table usage came from cases of pneumonia, tracheitis, and urinary tract infections. These cases do not have a pre-defined AE-specific NAESS table, so the study team had predicted use of the generic NAESS table. Instead, the reviewers chose to use either the “culture positive sepsis” or “culture negative sepsis” tables in the tool. Additionally, there was no AE-specific table for PDA in the current tool and some PDA cases were scored using AE-specific tables for “respiratory insufficiency” or “edema”.

## Discussion

This is the first study to evaluate the validity of the NAESS tool, demonstrating an intra-class correlation (ICC) of 0.63 which indicates moderate reliability [7]. The variation due to individual reviewers was very small, so there was no appreciable reviewer bias such as a tendency to grade high or low. The limitation of the NAESS tool, as used in this study, is due to other sources of variation or error including lack of detailed explanation of events on the AE case report forms, variation in use of Generic versus Specific NAESS Tables, and a study design where deaths were not routinely included. Additionally, unlike real-time AE severity grading, reviewers did not have access to the clinical team who cared for the neonates with information limited to data on case report forms.

The ICC of 0.63 is similar to that observed in other studies of AE reporting. A study of oncologists rating adverse symptom events in cancer patients demonstrated an ICC ranging from 0.46 for vomiting to 0.71 for neuropathy [8]. The Spinal Adverse Events Severity system (SAVES-V2) demonstrated an interobserver ICC of 0.75 in a retrospective study using clinical vignettes [9]. Our study is unique in its use of actual case report forms from an RCT. Site investigators and study coordinators with full access to neonates, their electronic health record, and their care providers would likely have resulted in a higher ICC. However, our study design using true CRF data is consistent with AE review processes at the sponsor, DSMB, and regulatory levels where information is limited to data contained in case report forms. It is possible that the ICC would be higher if a more homogeneous group of reviewers (either by job title or years of experience) were used, but this study was designed to assess the tool reliability in a diverse group of individuals. We understand that various stakeholders will be using this tool in neonatal drug development and purposefully recruited the reviewer pool to reflect this.

This study highlights areas for improvement in AE case report forms. Reviewers attributed difficulty in assigning a severity score due to the limited information provided on these standard AE case report forms. NAESS tables required reviewers to consider changes in age appropriate behavior, basal physiology and the treatments provided for events. Without such information, some variation in scoring is not surprising. AE forms offer blank spaces to name and describe the event often in a narrative format. Non-serious AE case report forms typically offer limited one-line space for each event such that multiple events are collected on a single page. This lack of information and inconsistent narrative approach makes it difficult to assign severity, particularly in premature neonates in whom the outcomes reflect multi-organ disease processes. The NAESS severity grading criteria could be embedded into standard case report forms so that site investigators would provide the assessment of minor or major changes in baseline age-appropriate behavior or basal physiologic processes. Case report forms could also specify specific treatments required for patient stabilization. For example, information about additional medications, procedures, or changes in respiratory support in response to the event would aid in severity assessment. Current prospective validation studies of the NAESS tool will permit reviewers to have access to rich real-time clinical information when assigning the severity score which should improve accuracy and reliability.

There were two ICC sub-analyses performed. In the first analysis, the ICC was higher for AEs compared to SAEs (0.72 vs 0.55). This was an unexpected result because the SAE forms had much more rich detailed information while the AE forms had minimal clinical details. Paradoxically, fewer details may actually have led to less subjectivity in scoring the AE. More serious AEs may have been harder to classify because of the complex information presented which could lead to some uncertainty in the score by the case reviewer. When all four raters used the exact same NAESS table to score the AE (40 raters), the ICC was 0.63 and when the raters used at least one different table, the ICC was 0.56. This implies that different raters using varying tables for the same AE could decrease the reliability of the tool and improving standard training around the use of the tool could also improve reliability. The NAESS tool is complex in that there is a generic table and multiple AE-specific tables and it is not known if less complexity of the tool would increase reliability. The AE-specific tables allow a lot more relevant details for a specific AE to be considered in severity scoring. In future studies, we plan to assess the trade-off of simplicity (less tables) for specificity (multiple AE-specific tables).

Study design also likely contributed to lower ICC. If more than one death had been included, the ICC would have been higher. In addition, many reviewers chose to use a NAESS table other than what had been recommended. If the study team had assigned specific NAESS table for each case, perhaps the ICC would be higher. This real-world exploration of the NAESS revealed inconsistency over which NAESS table to use for specific types of events. The varied use of Generic versus Specific NAESS tables was an interesting and unexpected finding that highlights the importance of this study in end-users who are naïve to the tool. The study team chose to leave some autonomy to case reviewers because we wanted the tool to be as “real-world” as possible during this first step of validation process. The NAESS tool could be improved by providing more explicit instructions and training on the tool and by the addition of even more event-specific tables. Standard event terminology could help trigger the appropriate NAESS tool and may also allow linkage between EHR and AE reporting [1].

Reviewers preferred specific AE tables to the generic table even when the event did not match an existing specific event table. For example, for cases of infections (pneumonia, tracheitis) some reviewers chose the specific table “culture negative sepsis” instead of the generic table. In a case of hepatic hemorrhage, some reviewers used the gastrointestinal bleeding Specific AE table instead of the generic table. The NAESS does not yet include specific tables for laboratory abnormalities. For a case of thrombocytopenia, the reviewer used “coagulation disorder” instead of the generic table. Although this first iteration of the NAESS tool contained 35 specific tables, it is now clear that more specific tables are needed. Along with further infectious disease related tables, a specific table for PDA and specific laboratory abnormalities could immediately improve the tool.

The seven cases that returned the most variable severity scores (three different severity grades among the reviewers) highlight the need for improvement and standardization in AE data collection and reporting. For these specific 7 cases, all reviewers used the same NAESS tables. More education and understanding about assessing basal physiology and using the NAESS may improve consistency. These cases were likely to have insufficient data for the reviewer to make a confident severity assessment and may reinforce the need to standardize the information collected on AE case-report forms, specifically treatments provided and changes in basal physiology. As discussed, there was greater consistency in severity scores for AEs compared to SAEs.

This study has strengths and limitations. The strengths of the study include the use of real case report forms from a recent neonatal RCT [4]. This use of real AE data allowed us to test the NAESS tool in a retrospective way that was internally validated. In addition, the 12 reviewers were naïve to the tool itself, so this study represented how the tool will perform in the hands of new users from multiple stakeholder groups. Lastly, this validation study captured a wide range of AE types and severity, allowing assessment over the entire range of the tool. The major limitation to this study was that the data collection was non-standardized and did not contain detailed information. The reviewers received copies of the case report forms, exactly the way the local study team documented. The reviewers commented that the major limitation of using the NAESS was the brevity of the information and inconsistency of detail provided on the case-report forms. In this way, this research study cannot purely assess rater performance because the cases themselves add some variability to the severity rating. This reinforces the need to standardize SAE/AE reporting and continue to strengthen the tool for more wide-spread use. Revisions in AE case report forms are needed to enhance data quality and severity assessment. A prospective validation study might overcome these limitations with the use of for purpose-designed case report forms and real-time bedside clinical information.

In addition to creation of this new NAESS tool, the neonatal clinical trial space needs a more standard and comprehensive way to collect AE data. Ongoing efforts at standardization of terminology and data harmonization [1, 10] will improve data collection and may even allow the EHR to systematically capture AEs. To facilitate this goal, the NAESS adverse event terminology is publicly available on the NCI Thesaurus (https://ev.nci.nih.gov/ftp1/INC/Adverse_Events_terminology) and terms have been mapped to NICHD Pediatric AE terminology and MedDRA lowest level terms. In the NICU, standardized minimum datasets have been used to improve research efforts and quality improvement with consensus groups having identified 12 critical outcomes for all future trials involving neonates cared for in high-income settings [11]. There is significant overlap in these critical outcomes and AEs, therefore uniform reporting of core-outcomes would also improve AE reporting. Leveraging the EHR in automated data-capture provides another mechanism to standardize AE reporting, with automated data capture from the EHR improved AE reporting for bacterial infections in children with acute myeloid leukemia [12]. With continued efforts to standardize terminology, data elements, and core outcome measures, we can leverage the EHR to improve AE reporting in neonates.

Improved processes of data collection and standardization of AE reporting are critical to patient safety and advancing neonatal therapeutics. The NAESS can be expanded to include more terms and conditions and specific education efforts on the use of the tool are being developed. Criteria for laboratory AEs are also needed as neonatal reference ranges are different from those in older patients. A prospective study using NAESS is ongoing, with the addition of standard training of case reviewers to address some of the limitations of this retrospective study.
